# A Randomized, Double-Blinded, Placebo-Controlled Study to Compare the Safety and Efficacy of Low Dose Enhanced Wild Blueberry Powder and Wild Blueberry Extract (ThinkBlue™) in Maintenance of Episodic and Working Memory in Older Adults

**DOI:** 10.3390/nu10060660

**Published:** 2018-05-23

**Authors:** Adrian R. Whyte, Nancy Cheng, Emilie Fromentin, Claire M. Williams

**Affiliations:** 1 School of Psychology and Clinical Language Sciences, University of Reading, Reading RG6 6AL, UK; a.r.whyte@reading.ac.uk (A.R.W.); nancy.cheng@pgr.reading.ac.uk (N.C.); 2 Naturex Inc., 375 Huyler Street, South Hackensack, NJ 07606, USA; e.fromentin@naturex-dbs.com

**Keywords:** wild blueberry, flavonoid, anthocyanin, cognition, blood pressure, episodic memory, older adults

## Abstract

Previous research has shown beneficial effects of polyphenol-rich diets in ameliorating cognitive decline in aging adults. Here, using a randomized, double blinded, placebo-controlled chronic intervention, we investigated the effect of two proprietary blueberry formulations on cognitive performance in older adults; a whole wild blueberry powder at 500 mg (WBP500) and 1000 mg (WBP1000) and a purified extract at 100 mg (WBE111). One hundred and twenty-two older adults (65–80 years) were randomly allocated to a 6-month, daily regimen of either placebo or one of the three interventions. Participants were tested at baseline, 3, and 6 months on a battery of cognitive tasks targeting episodic memory, working memory and executive function, alongside mood and cardiovascular health parameters. Linear mixed model analysis found intervention to be a significant predictor of delayed word recognition on the Reys Auditory Verbal Learning Task (RAVLT), with simple contrast analysis revealing significantly better performance following WBE111 at 3 months. Similarly, performance on the Corsi Block task was predicted by treatment, with simple contrast analysis revealing a trend for better performance at 3 months following WBE111. Treatment also significantly predicted systolic blood pressure (SBP) with simple contrast analysis revealing lower SBP following intervention with WBE111 in comparison to placebo. These results indicate 3 months intervention with WBE111 can facilitate better episodic memory performance in an elderly population and reduce cardiovascular risk factors over 6 months.

## 1. Introduction

The number of people aged 60 years or older is expected to rise from 900 million to 2 billion between 2015 and 2050, an increase from 12 to 22% of the global population [1]. From age 30 onwards, it is well documented that individuals are subject to increasing cognitive decline which in turn can have an impact on the general well-being, health, and financial status of the individual [2]. In particular, domains of reasoning, problem solving skills, attention, processing speed, working memory, and episodic memory become a significant societal problem as we age [3]. With levels of aging increasing in the population, maintaining health and wellbeing in the elderly population by enhancing or slowing decline in cognitive performance has become a major medical challenge.

In the past few decades, the scientific community has started to study and understand the impact of the Western diet on many non-communicable diseases and has demonstrated a clear beneficial relationship between healthy nutrition and cognitive aging. For example, deleterious dietary habits (overfeeding, high caloric/low dietary fiber diets or consumption of low antioxidant nutrients) and sedentary lifestyle, or emotional stress, have been reported as key environmental factors for brain disorders [4].

To date, a number of studies have observed protective associations between dietary polyphenols and the prevention of age-related chronic diseases such as cardiovascular disease (CVD), diabetes, cancers, osteoporosis and neurodegenerative diseases [4]. Blueberries are a particularly rich source of polyphenolic compounds and contain a wide range of flavonoids (anthocyanins, catechins, quercetins, proanthocyanidins) and phenolic acids (chlorogenic acids) [5,6]. Flavonoids, found in a variety of fruits, vegetables, and beverages, are increasingly recognized as promising compounds mediating a range of actions. Spencer [7] proposes that these involve the ability to protect vulnerable neurons, enhance neuronal function, and stimulate regeneration via interaction with neuronal intracellular signaling pathways involved in neuronal survival and differentiation, long-term potentiation (LTP), and memory. Indeed, recent evidence suggests that the beneficial effects of flavonoids involve decreases in oxidative/inflammatory stress signaling, increases in protective signaling, and neurohormetic effects leading to the expression of genes that encode antioxidant enzymes, phase-2 enzymes, neurotrophic factors, and cytoprotective proteins. Specific examples of such pathways include the sirtuin-FoxO pathway, the NF-κB pathway, and the Nrf-2/ARE pathway. Together, these processes act to maintain brain homeostasis and play important roles in neuronal stress adaptation. It can therefore be proposed that polyphenols have the potential to prevent the progression of neurodegenerative pathologies. Additionally, polyphenols have a well-documented role in vascularization, with recent evidence demonstrating the effect of blueberry polyphenols on acute improvement of endothelium-dependent flow mediated dilation which correlate with appearance of specific metabolites in plasma [8].

Together, these neurobiological and vascular changes result in both acute and chronic improvements in memory and learning in both animals [7,9,10,11] and humans, with effects occurring within 0–6 h as well as after repeated dosing [12,13,14]. In children, improvements have been observed acutely following consumption of 200 g of blueberries in delayed recalled accuracy of the Rey’s Auditory Verbal Learning Test (RAVLT) and in delayed word recognition of this same task following consumption of 15 and 30 g of wild blueberry powder (WBP) respectively [15,16]. More recently, Whyte et al. [17] manipulated cognitive demand in a modified attention network task finding that, following acute intervention with 30 g WBP, children performed more effectively on the more cognitively demanding trials of the task. This indicates that WBP intervention may be particularly effective where cognitive demand or difficulty is comparatively high, and participants are not able to perform the tasks at, or near, ceiling. In healthy older adults, 90-day supplementation with 24 g of freeze-dried blueberry powder demonstrated significant improvement in repetition errors in the California Verbal Learning Task (CVLT) and reduced switch cost on a task-switching test [18]. Linked to the findings of Whyte et al. [17], Miller at al., noted that their significant findings were found on the more challenging cognitive tasks in their battery. Similarly, in older adults with mild cognitive impairment (MCI), participants showed better episodic memory performance on the CVLT and Verbal Paired Associate Learning Test after 12-weeks consumption of 400–600 mL of wild blueberry juice [19]. In terms of neurological studies, 12-weeks supplementation with 30 mL daily blueberry concentrate showed significant resting state increases in grey matter perfusion and increased activity in brain areas significantly related to the cognitive tasks performed [20]. Additionally, Boespflug and colleagues [21] found increased brain activation in the pre-central gyrus, middle frontal gyrus, and parietal lobe during a working memory, n-back, task from 16 weeks blueberry supplementation, however no behavioral benefits were found.

Together this previous evidence suggests that the exposure to flavonoids, and blueberry flavonoids in particular, may be beneficial to episodic memory with some potential effects on other aspects of cognition, including executive function and working memory. However, more studies are needed to help firmly establish an association between flavonoid dose, duration of exposure, and cognitive performance [22]. Indeed, a recent systematic review concludes that the positive effects of food-based anthocyanin consumption, the primary flavonoid component found in berries, on both acute and long-term cognition appears promising [23]; and anthocyanins may be one of the components leading to the efficacy of blueberries on cognition and vascularization. However, anthocyanins have low bioavailability and, consequently, blueberry studies have typically had to utilize high doses that are impractical for daily human consumption and reduce compliance in long term studies. Furthermore, anthocyanins are relatively unstable in human body fluids at pH 7 and 37 °C, thus strategies to improve anthocyanin bioavailability and influence their bioactivity are highly desirable. Naturex Inc, has developed a formulation of wild blueberry extract and wild blueberry powder in combination with thiol-components (l-cysteine and l-glutathione) that reduce anthocyanin degradation at physiological conditions (patent number US7820207B2—Stabilized anthocyanin compositions).

The aim of this clinical study is to evaluate the safety and efficacy of daily wild blueberry extract and blueberry powder stabilized with l-cysteine and l-glutathione on episodic and working memory, executive function, mood and cardiovascular health over a 6-month period.

## 2. Materials and Methods

This study was reviewed and approved by the University of Reading Research Ethics Committee and was given a favorable ethical opinion for conduct (Project identification code UREC 15/18). All subjects gave their written informed consent for inclusion before they participated in the study. The study was registered at clinicaltrials.gov NCT02446314.

### 2.1. Participants

Based upon a medium effect size (*d* = 0.640) observed in previous studies using grouped executive function and episodic memory domains, we calculated that a sample size of 30 participants per experimental group would provide a power of 0.96. One hundred and twenty-two independently living healthy volunteers, 65–80 years old (mean = 70.8, SD = 3.88), of all ethnicity, with subjective self-reported memory complaints were therefore included in the study. 

Subjects were asked to maintain their normal eating habits and exercise habits to avoid any change in body weight over the duration of the study. At the screening/familiarization visit participants completed a number of questionnaires to check physical and mental health, and overall normal cognitive functioning. The Mini-Mental State Examination (MMSE) was used to give a measure of global cognitive function, the National Adult Reading Test (NART): to give a measure of premorbid intellectual functioning, the Consortium to Establish a Registry for Alzheimer’s Disease (CERAD): to give a measure of baseline cognitive function and Letter and category fluency: to measure aspects of language and executive retrieval functions. Volunteers with an MMSE <24 were not included in the study. Exclusion criteria included the use of complementary and alternative medicine (mostly food supplements) for memory or cognitive performance within 1 month prior to study participation, history of metabolic disorder, diabetes, substance abuse, diagnosis with psychiatric or neurological conditions, use of medications that might affect the outcome measures (such as antidepressant and sleeping medication), planned changes to current medication regime (treatment/dose), allergic reactions to compounds similar to those in the investigational product, participation in other clinical trials within the previous month or other cognitive trial within the previous 6 months, and drinking over 3 alcoholic beverages per day or more than 12 alcohol units per day. Additionally, participants completed a 3-day dietary record diary and YALE physical activity questionnaire in the seven days before the first and last visit. Demographic details of the participants are shown in Table 1.

### 2.2. Treatments

The study used a randomized, double-blinded, placebo-controlled study to assess the safety and efficacy of Wild Blueberry Powder and Wild Blueberry extract (ThinkBlue™, Naturex Inc, South Hackensack, NJ, USA) in maintenance of episodic and working memory in older adults (Table 2).

The investigational products were encapsulated in transparent capsules and delivered in blister packs of 16 capsules/week. The investigational products were manufactured by Naturex Inc and the capsules and blisters by US Pharmalab Inc. The participants were instructed to take 2 capsules daily and to return the left-over capsules by mail on a weekly basis to monitor compliance. The placebo powder (consisting of maltodextrin and food grade artificial dye—blue and red lake) was color matched to make these treatments indistinguishable, and to maintain blinding of both the investigator and the study subject. Randomization of participants to treatment group was carried out using a computerized random number generator (www.random.org) by Naturex Inc. Each subject entering the study was assigned a participant’s number which corresponds to one of the four groups. The relationship between the participant’s number and the treatment group was unknown to both the investigators and the participants, i.e., the study was double-blinded. Unblinding only occurred after the statistical analysis plan was signed and an analysis of the primary dependent variables was completed. 

### 2.3. Cognitive Function

E-prime V2 (Psychology Software Tools, Inc., Sharpsburg, PA, USA) was used to display stimuli and record participants responses. Audio stimuli were presented through noise cancelling enclosed headphones. Blood pressure and pulse were measured using OMRON M6 BP machines (OMRON, Hooffforp, Netherlands).

Both animal [9,11,24,25] and human [18,19,26,27,28] research has previously shown particular benefits for both visuo-spatial and verbal episodic memory following flavonoid rich interventions with older adults. Furthermore, in a review of acute flavonoid interventions, Bell [12] noted that one would expect episodic memory benefits in older adults over and above other cognitive domains. The primary outcome measures evaluated in the current study were therefore maintenance of episodic memory assessed via three episodic memory tasks completed at week 0, 12 and 24. These tasks targeted different visual and verbal components of episodic memory.

Firstly, the Rey’s Auditory Verbal Learning task (RAVLT) targeted verbal episodic memory and examined performance in learning, memory recall, and recognition. The procedure followed that previously described by Lezak [29], and as amended by Whyte, and Williams [17]. Measures of immediate recall, total acquisition, amount learned, proactive and retroactive interference, delayed recall, and word recognition were evaluated.

Secondly, visual episodic memory was assessed using an object recognition task. Here, 20 images were presented each for 1000 ms with a 200 ms inter-stimulus interval. Approximately 25 min later, these images were re-presented together with an additional 25 images (distractors). The subject was asked to identify whether the image is the original or a new one. All images were drawn from the grey scale version of the Snodgrass and Vanderwart picture set [30] and matched for familiarity, complexity, and imagery. 

Finally, we employed the Corsi Blocks task [31,32] as a measure of visual memory span [33] to target short term spatial episodic memory. In this electronic version of the task, participants were required to observe a sequence of blocks lighting up one at a time for a duration of 1000 ms. The participant then repeats the sequence back in the same order by pressing the relevant blocks on the screen. Sequence length was randomized and ranged from two to nine blocks. There were four versions of each length giving a total of 32 sequences. Key outcome measures are the number of correct sequences and the longest sequence remembered.

The secondary outcome measures evaluated working memory, selective attention and executive function. Working memory was evaluated via completion of two different tasks, which consisted of serial subtractions and Sternberg memory scanning. The serial subtractions tasks followed the same procedure as described previously by Bell et al. [34]. Average response time and accuracy were recorded.

The Sternberg memory scanning task [17,35] followed the same procedure as described previously by Bell et al. [34]. Reaction time and accuracy were recorded for each trial. Additionally, the coefficient of the line of best fit for reaction time versus memory set size was calculated to determine the scanning rate of the participant, whilst mean reaction time was represented by the intercept.

Executive function, including selective attention, was measured using the Modified Attention Network Task (MANT) and Stroop task. The MANT examines response interference, executive function and attention. The task followed the same procedure as previously described by Whyte et al. [17], with the exception that there were only two blocks each of which had a stimulus duration of 500 ms. Additionally, no noise condition was included. Accuracy and response time were measured separately.

The Stroop task examines response interference, executive function and attention. The task followed the procedure described previously by Bell et al. [34], with the exception that stimuli were displayed with no time limit for responding. Accuracy and response time were measured separately.

The Positive and Negative Affect Schedule—NOW (PANAS-NOW), a reliable and valid self-report measure [36,37], was used to determine positive and negative mood. Two scales, one for negative affect and one for positive affect, were calculated.

### 2.4. Markers of Cardiovascular Health

After the first half of the cognitive test battery, systolic and diastolic blood pressure, and heart rate were measured three times in a row with a two-minute gap between each measurement. OMRON M6 blood pressure machines (OMRON, Hooffforp, Netherlands) were used to take the measurements. Subjects were in a seated position with their back against the chair. Mean scores were calculated for each measure.

### 2.5. Food Diary

Participants completed two three-day food diaries with the initial diary completed between visits 1 and 2 and the second completed during the week before visit 6. Weighted food and drink was recorded for two weekdays and one weekend day. These diaries were used to calculate habitual fruit and vegetable, total flavonoid, and anthocyanin consumption at the beginning and end of the study. Flavonoid content of food diary items was drawn from the USDA Database for the Flavonoid Content of Selected Food [5]. Where food items were not represented in the USDA database, the Phenol Explorer database was used [38] or a literature search was conducted for recent relevant flavonoid content studies.

### 2.6. Procedure

The study comprised of six visits, including a screening/familiarization visit (week T-1), three test visits (week 0, 12 and 24), and two control visits (week 6 and 18). Following the 1-week screening period, subjects were randomized to consume either placebo, wild blueberry powder-500 mg, wild blueberry power-1000 mg or wild blueberry extract-111 mg. All eligible participants consumed their assigned capsules once daily orally with breakfast. On test days the capsules were consumed after the cognitive battery had been completed.

During each test day, the participants attended the University of Reading labs at 0800 and first consumed a standardized breakfast of two 40 g Pasquier Croissants, 25 g Philadephia Light cream cheese and water ad libitum. Weight, height and wellbeing were then measured along with checks on any changes to smoking, alcohol intake, medication, and any adverse effects of intervention. The tasks used in this study have previously been shown to be sensitive to flavonoid interventions and are suitable for use in this population. Participants performed the first five cognitive tasks; the Rey’s Auditory Verbal Learning Task (RAVLT), Picture Recognition Task, Corsi Block Task, Stroop Task, and Modified Attention Network Task (MANT). There was then a short break where blood pressure and pulse were recorded three times in a row with a 2-min gap between each reading. The final three cognitive tasks; the Serial 3 s, Serial 7 s, and Sternberg task were then performed, and then finally the measure of mood, using the PANAS-NOW. In total the test session took no more than two hours to complete. Multiple matched versions of the RAVLT and Corsi-Block task were constructed and the order of presentation to participants was randomized across test sessions using a balanced Latin square.

The control visits consisted of a check on wellbeing along with checks on any changes to smoking, alcohol intake, medication, and any adverse effects of intervention. Following each test and control visit, capsules for the following 6 weeks were administered.

### 2.7. Statistical Analysis

In order to account for familywise error, and according to a strict a-priori statistics plan, analysis was limited to a restricted number of cognitive and cardiovascular outcomes (as detailed above in task descriptions). All analysis was performed using SPSS v22 (IBM, Armonk, NY, USA) on a per-protocol basis. Linear mixed modelling (LMM) was used to analyze cognitive and cardiovascular data employing an unstructured matrix to model successive repeat measurements. Session, Dose, and Session by Dose interaction were included as fixed factors in the model with baseline performance being included as a covariate. In the event of a significant interaction, data was split by time point and two further one-way LMM analyses of Dose with baseline performance included as a covariate were performed. Simple contrast analyses, with placebo as reference, were used to investigate any significant main effects found in the above analyses. Outliers were removed based on comparison to Standard Deviation (SD > 2SD) at each time point for each group. Given there were only two sets of records, food diaries were analyzed employing an unstructured matrix to model successive repeat measurements at time points one and six. Time point, Dose, and Time point by Dose interaction were included as fixed factors.

## 3. Results

**Figure 1:**
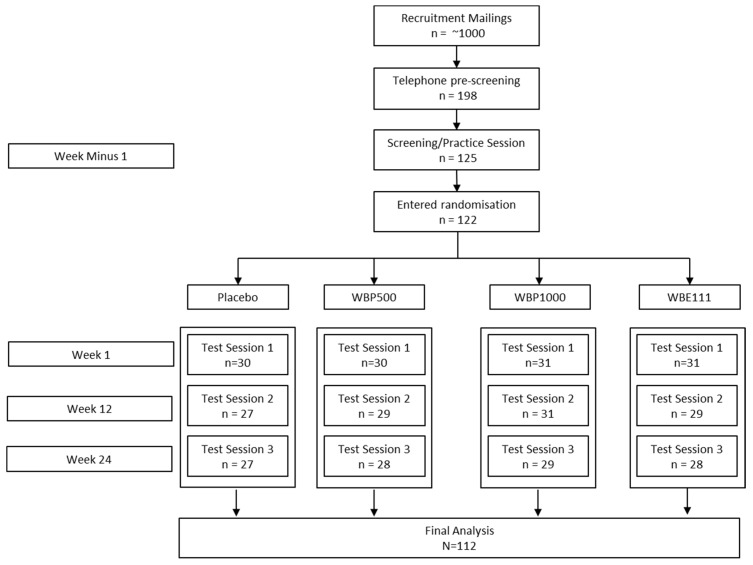
Treatment allocation and participant numbers throughout study.

One hundred and twenty-two adults aged between 65 and 80 years were randomly allocated to a 6-month, daily regimen of either placebo, 500 mg wild blueberry powder formulation (WBP500), 1000 mg wild blueberry powder formulation (WPB1000), or 111 mg wild blueberry extract formulation (WBE111). One hundred and fifteen participants completed the full course, however, a further three were subsequently excluded from the analysis, two were excluded due to beginning a course of antidepressants during the study, and one for non-compliance in consumption of the capsules. Participant numbers at each stage of the study are shown in Figure 1 below.

### 3.1. Episodic Memory Outcomes

#### 3.1.1. Word Recognition—Proportion of Words Recognized Following a 20 Min Delay

**Figure 2:**
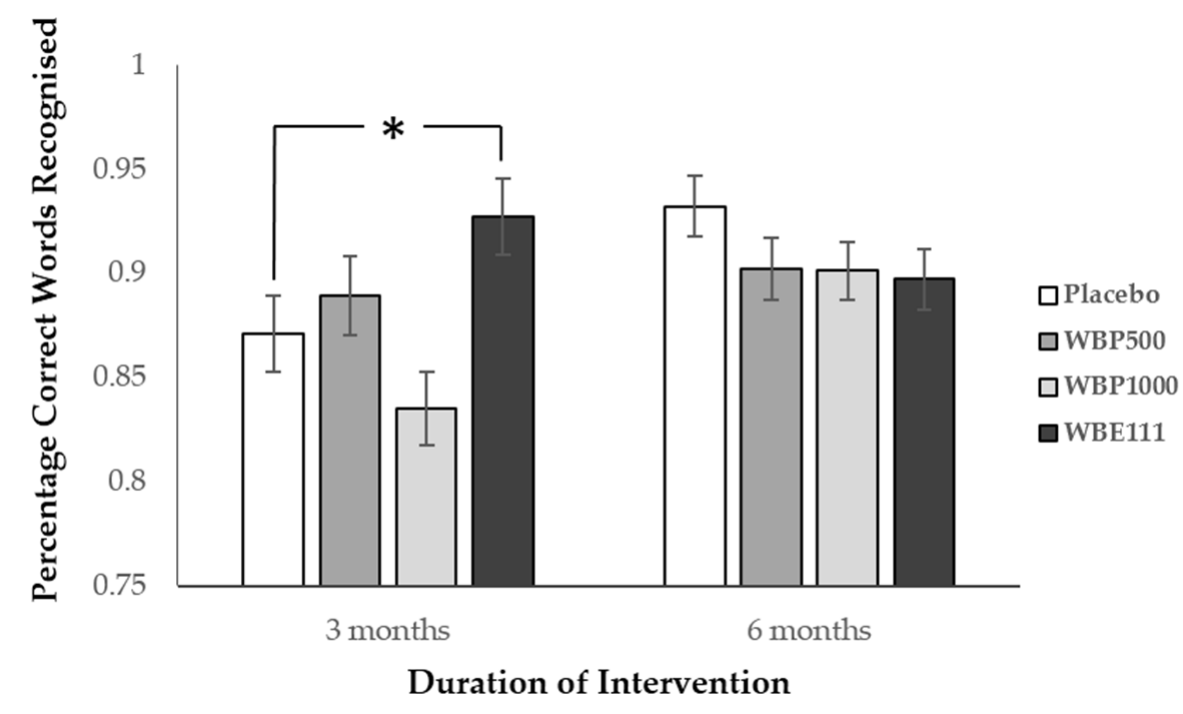
Word recognition performance (±SE) by intervention at 3 and 6 months. Performance for WBE100 was significantly better than placebo (* *p* < 0.05) following 3 months of intervention, but not at 6 months.

The data from 15 participants who were outliers was excluded from this analysis. There was a significant effect whereby session significantly predicted performance (*F*(1,97) = 7.40, *p* = 0.008). Importantly there was also a significant intervention by session interaction (*F*(3,97) = 4.99, *p* = 0.003). Data was therefore split by session and two one-way analyses were performed. For the Session 2 analysis, intervention significantly predicted performance (*F*(3,92) = 4.4, *p* = 0.006). As shown in Figure 2, Post hoc simple contrast analysis, using placebo as the reference category, revealed a significantly better performance following WBE111 intervention (mean = 0.926) in comparison to Placebo (mean = 0.871) (*p* = 0.038). There were no significant effects at session 3.

#### 3.1.2. Corsi Block—Total Number of Sequences Correctly Recalled

**Figure 3:**
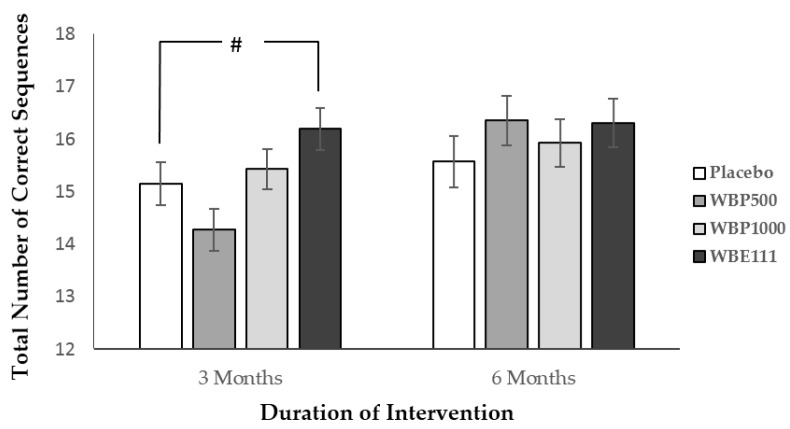
Total number of correct sequences recalled (±SE) by intervention at 3 and 6 months. There was a trend for better WBE100 performance in comparison to placebo (# *p* < 0.07) following 3 months of intervention, but not at 6 months.

The data from 11 participants who were outliers was excluded from this analysis. Here, session significantly predicted performance (*F*(1,101) = 12.3, *p* = 0.001) and a significant intervention by session interaction (*F*(3,101) = 3.95, *p* = 0.010). Data was therefore split by session and two one-way analyses were performed. For the Session 2 analysis, intervention significantly predicted performance (*F*(3,101) = 4.05, *p* = 0.009). As can be seen in Figure 3, post hoc simple contrast analysis, using placebo as the reference category, revealed a trend towards better performance following WBE111 intervention (mean = 16.19) in comparison to Placebo (mean = 15.14) (*p* = 0.069). There were no significant effects found at session 3.

There were no other treatment related significant effects or interactions for the Episodic Memory analyses.

### 3.2. Markers of Cardiovascular Health

#### Systolic Blood Pressure (SBP)

**Figure 4:**
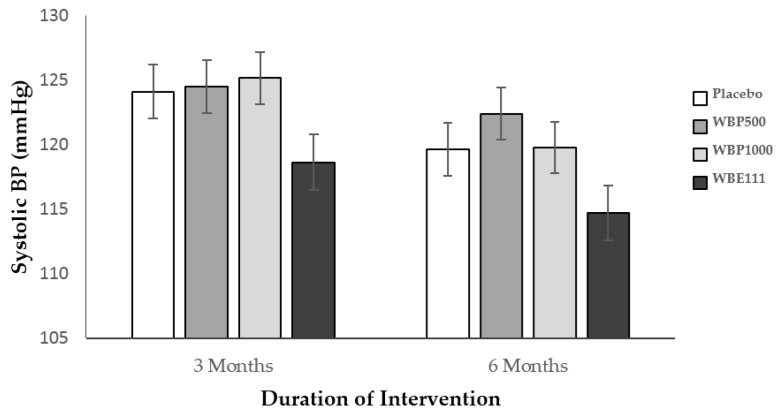
Systolic blood pressure (±SE) by intervention showing a main effect of WBE111 intervention in comparison to placebo over 3 and 6 months.

The data from ten participants who were outliers was excluded from this analysis. There was a significant effect whereby session predicted SBP (*F*(1,102) = 12.9, *p* = 0.001). Importantly, intervention also significantly predicted SBP (*F*(1,102) = 2.88, *p* = 0.040). Post hoc simple contrast analysis, using placebo as the reference category, revealed lower SBP following WBE111 intervention (mean = 116 mmHg) in comparison to Placebo (mean = 122 mmHg) (*p* = 0.039). For WBE111, this equates to a mean reduction of −3.08 mmHg between baseline and 3 months, and −6.99 mmHg between baseline and 6 months, whereas for placebo there was an increase of 1.73 mmHg between baseline and 3 months, and a decrease of only −3.19 between baseline and 6 months. As there was no significant interaction, no further one-way analyses were performed. The mean scores for SBP by group and time point are shown in Figure 4. 

There were no other treatment related significant effects or interactions for the markers of cardiovascular health.

### 3.3. Working Memory, Executive Function, and Mood Outcomes

There were no other treatment related significant effects or interactions for the remaining analyses.

### 3.4. Food Diary Analysis

In total, 283 dietary items classified as fruit or vegetables, and/or with flavonoid content, were identified. A summary of mean fruit and vegetable, total flavonoid, and anthocyanin content is shown in Table 3 below. No significant main effects or interactions were seen on any measure indicating there was no change to fruit and vegetable, total flavonoid, or anthocyanin consumption in any group over the 6-month duration of the study. 

## 4. Discussion

This is the first randomized double-blind, placebo-controlled study demonstrating an efficacy of a proprietary formulation of blueberry containing as low as 50 mg of polyphenols, comprising anthocyanins, flavanols, proanthocyanidins and chlorogenic acids (WBE111). Here we have shown improved episodic memory performance in delayed word recognition and marginally significant improved visuo-spatial Corsi Block performance at 3, but not 6, months following intervention alongside a lowering of systolic blood pressure at both time points. No such effect was found for the wild blueberry powders (WBP500 and WBP1000). Furthermore, no difference between groups or between time points was found on any measure of fruit and veg, total flavonoid, or anthocyanin consumption giving a clear indication that habitual diet was maintained over the six months of the study.

It is of particular interest that the beneficial cognitive effects of our intervention were found within the domain of episodic memory. This is in keeping with previous polyphenol-rich, chronic blueberry interventions with older adults which have found positive memory related benefits on the CVLT, a task similar to the AVLT used in the present study [18,19]. The fact this finding was for word recognition is important as research from our lab has shown this task to be particularly sensitive to flavonoid rich berry intervention in older adults [28] and children [15]. As was the case with the current study, these effects were found in the absence of significant benefits in other memory measures. It has been proposed that the cognitive benefits of flavonoid intervention are often found when the task is cognitively challenging especially where stimuli are presented with some degree of interference [17,18]. Word recognition requires that participants discern previously presented words from an initial list whilst disregarding words from a second list and further novel interference words all of which have been phonetically or semantically matched. Given the interference element of this task, and taking previous findings into account, it can therefore be proposed that recognition memory is particularly sensitive to flavonoid intervention. Though our visuo-spatial findings here did not quite reach significance, beneficial effects of blueberry supplementation on visuo-spatial memory in aging rats is well documented (for example [9,25]), and polyphenol-rich chronic interventions with procyanidins have previously shown positive visuo-spatial benefits for older adults [26,27]. Importantly, the episodic verbal and visuo-spatial effects found in the current study were achieved at a much lower blueberry anthocyanin dose (7 mg) than those used with older adults in previous studies where typical doses were in the range of 269–621 mg daily [18,19,20,21]. This indicates that, due to the stabilizing properties of the current formulation, it is possible to facilitate beneficial cognitive effects with relatively low daily anthocyanin doses.

No positive effects were found for the executive function and working memory domains considered in the current study. In a review of the polyphenol literature [13], Lamport and colleagues note that, with the exception of isoflavones, positive effects of intervention are most often found within the domain of declarative memory (though see [18,20]). Furthermore, in a review of the acute flavonoid literature, Bell [12] found that, whilst executive function benefits might be found in young adults, this was not the case for older adults who instead demonstrated most reliable improvements in episodic memory. Given that it is well documented that episodic memory performance is known to decrease in older individuals; the findings in the current study indicate that this domain may be particularly sensitive to polyphenol rich interventions within an older age group. This is consistent with previous findings which have shown elevated or sustained levels of Brain Derived Neurotropic Factor (BDNF) in ageing rats [9] and humans [28,39] following flavonoid-rich interventions. BDNF is known to be involved in hippocampal function related to episodic memory and may enhance neuronal connections or neuronal growth [12] leading to the improved memory performance demonstrated here.

The positive effects on cognition of lowering blood pressure in the elderly are well documented [40] with recent reviews indicating that antihypertensive medication, particularly Angiotensin II receptor blockers, benefit episodic memory in elderly individuals [41,42]. Though it should be noted that our statistical plan was quite conservative in that it required the a-priori removal of outliers to 2 SD from the mean, our findings are consistent with this previous research in that the WBE111 both reduced systolic blood pressure and showed improved episodic memory at 3 months. Additionally, previous research has shown that, following acute wild blueberry intervention, increases in flavonoid metabolites coincide with increased flow-mediated dilation [8] and both acute [28] and chronic [20] blueberry interventions have shown increased resting state perfusion in younger and older adults respectively. Interestingly, again following chronic wild blueberry interventions, increased BOLD activation can be found in task related brain areas for both executive function Stroop [20] and working memory N-Back [21] tasks. Though, as yet, no wild blueberry study has considered brain activation in relation to episodic memory, our findings relating to improved vascular function indicate that one possible mechanism by which the WBE111 intervention is having an effect is via increased perfusion in task relevant areas such as the hippocampus.

As noted above, there was lack of any significant WBP-related cognitive or cardiovascular effects. One possible explanation for this finding may be the different flavonoid and polyphenol content of each treatment. Though the WBP1000 treatment contained a higher total polyphenol content (70 mg) in comparison to WBE (50 mg), the total WBP anthocyanin content (2.7 mg) was lower than WBE (7 mg). The WBP500 treatment was lower than WBE111 in both total polyphenol (35 mg) and anthocyanin (1.35 mg) content. Given the growing body of research relating flavonoid interventions to improved cognition [12,13,14] it is probable that anthocyanins may be a critical component in the positive effects found here. The lack of positive effects found for WBP500 and WBP1000 may therefore be due to the lower anthocyanin dose in these treatments not being sufficient to produce a beneficial effect. Further research considering doses equivalent or higher to the WBE111 treatment is therefore recommended. Alternatively, the microstructure of the extract in comparison to the powders should also be considered. The extract is devoid of fibers and water soluble while the full spectrum powders are very rich in insoluble fibers to which the polyphenols are bound. It is therefore possible to hypothesize that the polyphenols in the powders were not as bioaccessible as in the extract, and therefore less efficacious. It should be noted that Miller et al. (2017) found improved cognitive function following a full spectrum intervention with older adults, albeit at a much higher dose than those used here. It is therefore recommended that future research considers the effect of composition and dose of treatments to ascertain whether cognitive improvements can be achieved at lower anthocyanin doses in relation to level of fiber content.

The lack of a significant difference between the treatments at 6 months was unexpected. It is possible that the 6-month findings may indicate an element of practice effect whereby participants improved their strategy on performing these memory tasks thus reducing task sensitivity to intervention. Indeed, regardless of treatment, significant main effects of session were seen for both Corsi and Word Recognition indicating a general improvement in performance of these tasks across time. It should be noted that previous blueberry intervention fMRI research has found increased brain activation during cognitive tasks which was not mirrored in improved task performance again indicating a lack of task sensitivity to intervention [20,21]. It could therefore be argued, the fact that reduced systolic blood pressure was maintained for the WBE111 treatment in comparison to all other treatments at 6 months whilst the cognitive benefit was no longer evident is consistent with this task sensitivity hypothesis. 

Furthermore, the lack of significant findings at 6 months may be due to possible degradation of the active constituents in the capsules, or increased tolerance to the WBE, leading to a decrease in efficacy nearing the end of the study. This in turn may have reduced any differences between the interventions at this second time point. Analysis of the capsule contents were not measured during the current study, nor were any physical measures of flavonoid metabolism taken, precluding any definitive conclusions regarding these potential confounds. However, research should consider these as additional measurements across the duration of future studies.

Finally, there was a lack of mood effects found at any time point during the study. The mood measurements were taken at the end of a 1.5 h task session. Participants by this point were mentally fatigued and also could conceivably have been affected by their perception of how they had performed on the demanding task battery. This may in turn have confounded any positive or negative mood effects related to the interventions. To control for this, future research should consider measuring mood both before and after the task battery allowing for comparisons to be drawn on mood change between and during test sessions.

## 5. Conclusions

The results indicate that 3 months intervention with WBE111 can facilitate better episodic memory performance and improve cardiovascular function over 6 months. The doses used in this study were comparatively small compared to previous research and it is therefore interesting to see an effect even at such low dose. Effects were not found, however, for the working memory, executive function, and mood tests, and further research investigating the efficacy of interventions on these domains with higher daily doses is currently in train.

## Figures and Tables

**Table 1:** Demographic details of the participants.

Characteristic	Mean	SD	Range
Age (at enrolment)	70.8	3.88	65–80
Males	47		
Females	75		
Weight (kg)	70.7	3.88	42.5–118.9
Height (m)	1.68	0.09	1.49–1.88
Average Daily Alcohol Units	1.3	1.51	0–10
MMSE	27.1	1.65	24–30
NART (Number of Errors)	9.3	6.49	0–38
CERAD (Learning)	20.9	3.14	10–29
CERAD (Delayed Recall)	5.6	2.22	0–10
CERAD (Word Recognition)	9.55	0.93	3–10
CERAD (Word Rejection)	9.75	0.71	6–10
Letter Fluency	16.8	5.09	6–30
Category Fluency	18.9	4.95	9–32
Frequency of Forgetting 1	5	0.76	2.8–6.9
Frequency of Forgetting 2	5.22	1.04	2.4–7

**Table 2:** Nutritional composition of the three intervention products.

		Group			
		Placebo	Wild Blueberry Powder—500 mg	Wild Blueberry Powder—1000 mg	Wild Blueberry Extract 111 mg Thinkblue™
Intervention	Wild Blueberry Powder (mg/dose)	0	450	900	0
	Wild Blueberry Extract (mg/dose)	0	0	0	100
	l-Cysteine (mg/dose)	0	45	90	10
	l-Glutathione (mg/dose)	0	5	10	1
	Artificially colored Maltodextrin (mg/dose)	1000	500	0	889
Nutritional Analysis	Proteins (%)	1.0	8.4	15.8	10.5
	Fat (%)	5.0	6.1	7.2	0.8
	Fiber (%)	0.4	25.8	51.2	0.8
	Sugars (%)	2.6	7.6	13.2	1.2
	Vitamin C (ppm)	0.67	3.88	1.0	0
	Total Anthocyanin (mg/dose)	0	1.35	2.7	7
	Total Polyphenol (mg/dose)	0	35	70	50

**Table 3:** Mean daily Fruit and Vegetable (F and V), Total Flavonoid, and Anthocyanin (SD in parenthesis) consumption by dose at visits 1 and 6 of study.

	F and V (Portions)		Flavonoid (mg)		Anthocyanin (mg)	
Visit	1	6	1	6	1	6
Placebo	4.22 (2.14)	4.23 (2.44)	802.40 (625.16)	762.63 (670.12)	27.14 (30.64)	29.33 (36.90)
WBE110	3.40 (1.75)	3.46 (1.86)	859.41 (512.89)	739.65 (564.32)	22.47 (35.12)	19.18 (21.33)
WBP500	3.47 (1.57)	3.60 (2.01)	756.65 (546.53)	755.03 (576.46)	33.59 (46.45)	33.92 (45.41)
WBP1000	4.19 (2.57)	3.98 (2.73)	789.47 (514.20)	753.97 (476.98)	28.96 (28.96)	22.80 (30.21)
